# A new perspective on aqueous electrolyte solutions†

**DOI:** 10.1039/d5cp01781e

**Published:** 2025-07-15

**Authors:** Gerhard Schwaab, Simone Pezzotti

**Affiliations:** a Department of Physical Chemistry II, Ruhr-University Bochum Bochum Germany gerhard.schwaab@rub.de simone.pezzotti@ens.psl.eu +49 234 3224256

## Abstract

Aqueous electrolyte solutions are central to many natural phenomena and industrial applications leading to continuous development of increasingly complex analytical models. These are based on an atomistic description of electrostatic interactions between ions, along with mean-field approaches for the dielectric response of water. Despite many achievements, such concepts often fall short in quantitatively describing scenarios where ion–ion correlations and specific solvation effects become relevant, particularly in concentrated electrolyte solutions. Here, we propose a shift in perspective, by introducing a statistical, coarse-grained approach to describe the average thermodynamic properties of aqueous electrolyte solutions. This method eliminates the need to define ion pairs or ion complexes and does not require any prior knowledge on specific solvation. We base our concept on separating the solution into a spherical observation volume whose size and average composition are uniquely determined by the solution parameters, and its environment, which consists of the remaining solution. This separation allows us to express the volume–environment interaction in terms of a generalized multipole expansion, *i.e.* in a convenient, additive way. We applied this approach to 135 electrolytes including some notoriously complex species, such as LiCl or ZnCl_2_ over their full solubility ranges. This paves the road toward understanding super-saturated and water-in-salt solutions and electrolyte nucleation.

## Abstract 2

Concentrated electrolyte solutions present challenges for scientific research and technical applications. Here, we demonstrate that a small set of physically defined parameters can fully describe electrolyte and water activity.

## Graphical abstract

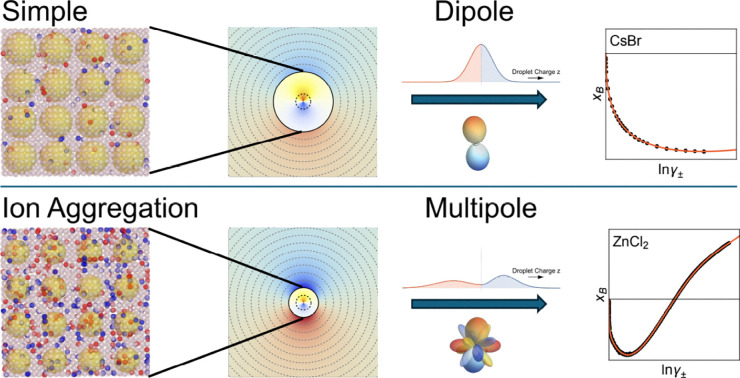

## 1 Introduction

A variety of technological challenges such as the understanding of water in salt electrolytes (WISE),^1–4^ the development of advanced battery and energy storage technologies,^5–9^ the recycling of desalination brines^10^ and a save operation of deep-sea boreholes^11^ require a thorough microscopic understanding of concentrated electrolyte solutions. However, existing heuristic descriptions of their average excess thermodynamic properties have been derived by extrapolating from the diluted regime, leaving a gap of knowledge for concentrated solutions.^12^

From a physicochemical perspective, the osmotic and average activity coefficients of electrolytes, *ϕ* and ln *γ*_±_, respectively, determine the excess thermodynamic functions of the corresponding aqueous and non-aqueous solutions.^13,14^ Debye and Hückel were the first to describe electrolyte and water properties in dilute electrolyte solutions as a function of ion concentration and electrolyte composition.^15^ The theory has later been extended to higher concentrations by Bjerrum, Glueckauf, McMillan, and Mayer (see review by Vaslow^16^). Friedman, Pitzer, and coworkers extended the description to more complex electrolyte mixtures such as seawater.^17^ A special issue of the Journal of Fluid Phase Equilibria^18^ celebrates the 100th anniversary of the findings by Debye and Hückel and provides a summary of recent developments on the topic. In that issue, Simonin and Bernard^19^ compare several simple activity models including Debye–Hückel, the mean spherical approximation, and the Pitzer approach. Earlier, Khan *et al.*^20^ compared four physical descriptions of ln *γ*_±_. While the Pitzer and Bromley approach accurately describes the activity coefficients of 1 : 1 electrolytes, the method fails for several important 1 : 2 electrolytes, such as CaCl_2_ and MgCl_2_. A recent review by Held^12^ provides a critical comparison of the different excess Gibbs energy parametrizations up to high electrolyte concentrations.

Common to all these descriptions is a series expansion of ln *γ*_±_ in the molality, the molarity, or the ionic strength framework starting from the limit of infinite dilution. In most cases, the dilute limit is described by a Debye–Hückel term. Each series expansion takes into account individual ion–ion interactions over the full volume in the configuration integral. The behavior at high molalities is in general derived by assuming ion-specific (hydrated) ion radii which are fitted to reproduce the experimental activity coefficients.^17,19^ The ability of ions to form different types of ion pairs or, more general, ion complexes makes an evaluation at high concentrations difficult.

Recently, a combined X-ray and simulation approach^21–23^ showed that at high concentrations, beyond the so-called Kirkwood-transition, ion–ion-correlations increase due to clustering, and the Debye length is no longer the characteristic length-scale. The critical concentration for the Kirkwood transition depends strongly on the electrolyte composition.

In spite of these complexities, it is very surprising that many experimental observables of aqueous electrolyte solutions, such as the effective molar extinction coefficients^24^ or the average apparent molar volume, show a nearly linear mol fraction dependency.^25^ This simple behavior suggests that, on a macroscopic level, most of the water-mediated ion–ion interactions partially compensate and lead to average interactions that can be described by simple analytical functions.

In the following, we focus on two-component solutions composed of a single electrolyte and water. We demonstrate that the separation of the solution into a well-chosen, uniquely defined, probe volume and its environment leads to a generalized multipole description of the excess interaction energy of electrolyte solutions. The number of required expansion orders is small: even for complex electrolytes such as ZnCl_2_ and LiCl three components are sufficient to describe the experimental data. When integrated, the resulting equation yields an analytical form of the osmotic coefficient *ϕ* and, thus, the water activity *a*_w_. The set of equations is applied to a total number of 135 electrolytes. The dependency of the fit parameters on electrolyte composition is discussed.

## 2 Introducing the statistical approach

**Fig. 1:**
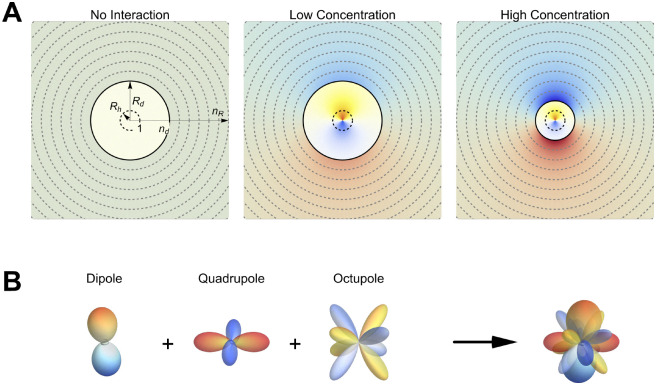
Schematics of the coarse-grained approach used to determine the interaction energy in aqueous electrolyte solutions. (A) The solution is separated into a uniquely defined central “observation volume” with radius, *R*_d_ (black line), and its environment. All distances *R* = *n*_*R*_*R*_h_ are measured in units of the hydrated electrolyte radius, *R*_h_ (dashed circle at the center), so that *n*_d_ = *R*_d_/*R*_h_ identifies the volume's radius in this coordinate system. In a hypothetical solution without ion–ion interactions, the charge distribution inside and outside the volume is uniform. When switching on ion–ion interactions, the solution averaged charge distribution inside the central volume produces an electrostatic potential in the local environment which attracts charges of the opposite sign. With increasing electrolyte concentration, *R*_d_ decreases, ion–ion interactions become more complex, and the interaction energy increases. (B) Example how, at high concentrations, dipole, quadrupole, and octupole interactions contribute to a complex interaction pattern between the observation volume and its environment. Please note that the charge distributions inside and outside the observation volume include both the ionic and the water contributions.

The mean activity coefficient expresses the excess chemical potential of the electrolyte in units of *RT*. Activity coefficients, *γ*_±_, as a function of molality, *m*_B_, are reported for a large number of salts in two books by Lobo.^26^ To describe *γ*_±_ based on a microscopic picture, our idea is to split the total electrolyte system into a spherical “observation” volume (d) with radius, *R*_d_, and the remaining environment (e). The excess chemical potential is hence a function of the interaction free energy, *U*_de_, between the two (see Fig. 1). We choose the observation volume to contain exactly one electrolyte unit (*ν*_+_ cations and *ν*_−_ anions) and the stoichiometric amount of water (*i.e.*, the number of water molecules per *ν*_+_ cations and *ν*_−_ anions)

1 *N*_w_ = *x*_w_/*x*_B_

where *x*_B_ and *x*_w_ are the electrolyte and water mol fractions, respectively. This definition is uniquely determined by the molality of the solution and its density. The spherical shape is adopted due to the isotropic nature of bulk electrolyte solutions.

This observation volume is illustrated in Fig. 1A. The left scheme pictures a hypothetical solution without ion–ion interactions, where the charge distribution inside and outside the probe volume is uniform. In the low concentration limit (center frame), the presence of ion–ion interactions causes a non-homogeneous charge distribution that polarizes the surrounding environment. As discussed thereafter, this is well described by dipolar interactions (see Fig. 1B). With increasing concentration, the radius of the probe volume decreases due to its stoichiometric definition, while the charge distribution inside and outside the volume becomes increasingly complex and requires higher-order multipole interactions.

It is important to note that our observation volume is a purely statistical entity, defined to be charge neutral and containing on average the smallest possible, stoichiometric number of water and ions. Hence, its size, charge, and average composition are unrelated to the important spatial features, such as inhomogeneities in ion distribution and ion pairing, and the consequences of these on the water network observed, *e.g.*, from simulations or diffraction experiments. However, the heterogeneity in the distribution of such configurations contributes, in our approach, to the instantaneous charge distribution inside the observation volume, due to both ions and water. This is a key feature of our approach, since water actively contributes to the charge distribution within the volume. This contribution is expected to be essential at high concentration, where specific ion solvation and water network arrangements dominate the free energy changes. These effects are, hence, naturally included in our charge distributions, which can be complex for strongly interacting ions where long-range ion–ion correlations and ion-clustering become important (see Fig. 1B).

The resulting charge distribution generates an electrostatic potential in the environment of our observation volume. The average interaction of this potential with the charge distributions outside the observation volume (which also depends on both ions and water), averaged over all possible configurations explored by the system, determines the interaction energy *U*_de_. We choose to describe such an electrostatic potential at a distance, *R*, outside the volume as a multipole expansion in spherical coordinates. We express this distance, *R* = *n*_*R*_*R*_h_, in units of the hydration radius, *R*_h_, of the electrolyte. It is determined by the composition of the solution, its density at constant temperature and pressure, and an effective number *N*_h_ of hydration water that depends on the electrolyte and the expansion order (see Appendix for details). This choice allows us to conveniently express the interactions in terms of a dimensionless distance unit, *n*_*R*_, removing the dependence on concentration and molar volume.

In a second step, we express the angular dependency of the effective charge at a distance *R* > *R*_d_ in terms of spherical harmonics (see Appendix for details). Thus, for each multipole term of order *l*, the integration over the angles leads to a contribution *U*_de,*l*_(*n*_*R*_) = *U*_*l*_*w*_*l*_(*n*_*R*_) where *U*_*l*_ is the *l*th order interaction energy. The weighting function, *w*_*l*_(*n*_*R*_), describes the radius dependency of the interaction strength. The total interaction energy of the observation volume with its environment is given by2
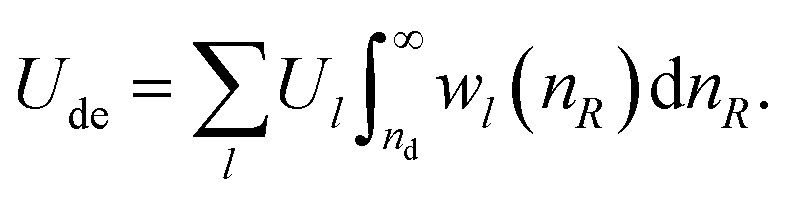


Please note that the integration starts at the volume boundary (*n*_d_ = *R*_d_/*R*_h_), which is solely determined by the composition of the solution and the hydration shell size of the electrolyte and is always positive.

The weighting function *w*_*l*_(*n*_*R*_) must satisfy the following conditions: (a) it must take into account the volume effect, *i.e.*, the increasing number of charges interacting with the probe volume (b) the interaction strength due to the screening between the volume surface and *n*_*R*_ must decay rapidly enough for eqn (2) to be integrable (c) it must take into account the fact that the charge distribution outside the probe volume is not purely random due to charge–charge interactions and hydrogen bonding, which limits the screening efficiency. (d) Different interaction orders require different screening lengths (e) all orders must vanish towards infinite dilution (*i.e. n*_d_ → ∞), (f) its integrated form must be consistent with the experimentally observed activity coefficients. We found heuristically (see Appendix for details) that a weighting function of the form3
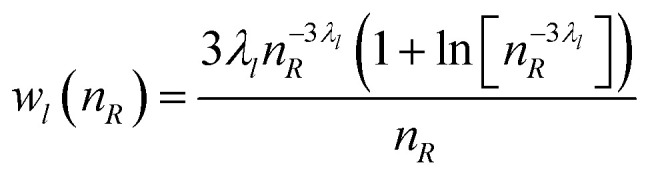
fulfills all these requirements and allows for a good representation of the activity coefficients for 135 electrolytes in their whole solubility range. By adopting this, the integration yields4
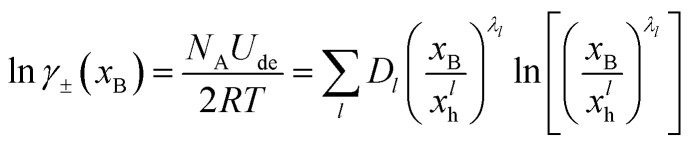
where we have converted the microscopic interaction energy *U*_de_ to molar quantities and normalized by the thermal energy *RT*. In addition, we have made use of the fact that

5 *n*_d_^3^ = (*R*_d_/*R*_h_)^3^ = *x*_h_/*x*_B_

defines a ratio of the mol fraction of the hydrated electrolyte, *x*_h_ and *x*_B_. This quantity is inversely proportional to the ratio of the observation volume and volume of the hydrated electrolyte, and, thus, to their mol fraction ratios (see Appendix for details). *D*_*l*_, *x*^*l*^_h_, and *λ*_*l*_ are electrolyte dependent fit parameters. *D*_*l*_ characterizes the depth of the *l*th order interaction energy profile. The parameter *x*^*l*^_h_ describes the crossover from the dilute to concentrated solutions. For *x*_B_ > *x*^*l*^_h_, the *l*th order contribution becomes positive, indicating unfavorable (endothermic) interaction. We found in our analysis (see figures below and Tables 1 and 2) that solely the dipolar (*l* = 1) contribution requires in some cases *x*^1^_h_ < 1. In all fits where quadrupole and octupole contributions become relevant, *x*^*l*>1^_h_ = 1 leads to a satisfying description of the experimental data.

**Table 1:** Fit parameters for ln *γ*, part 1

Salt	*x*_h,Dipole_	*D*_Dipole_	*λ*_Dipole_	*D*_Quadrupole_	*λ*_Quadrupole_	*D*_Octupole_	*λ*_Octupole_
NH_4_Br	0.348(26)	1.453(7)	0.517(12)	—	—	—	—
NH_4_Cl	0.370(9)	1.586(3)	0.537(4)	—	—	—	—
(NH_4_)_2_HPO_4_	1	8.866(25)	0.5422(12)	—	—	—	—
NH_4_NO_3_	1	2.840(23)	0.5591(22)	5.2(5)	1.91(6)	—	—
NH_4_ClO_4_	1	3.234(26)	0.576(3)	—	—	—	—
(NH_4_)_2_SO_4_	1	6.10(3)	0.4969(31)	—	—	—	—
NH_4_SCN	1	2.025(8)	0.5076(17)	—	—	—	—
BaBr_2_	0.05334(20)	2.287(5)	0.5449(16)	—	—	—	—
BaCl_2_	0.0836(15)	2.607(8)	0.527(3)	—	—	—	—
BaOH_2_	0.071(20)	3.27(18)	0.586(17)	—	—	—	—
BaI_2_	0.02945(9)	1.848(8)	0.5648(27)	—	—	—	—
Ba(NO_3_)_2_	1	6.72(10)	0.516(4)	—	—	—	—
Ba(ClO_4_)_2_	0.04917(11)	2.122(7)	0.5292(29)	—	—	—	—
CdBr_2_	0.926(18)	9.843(15)	0.4500(13)	—	—	—	—
CdCl_2_	0.034(7)	4.47(24)	0.597(12)	70(10)	1.354(13)	—	—
CdI_2_	0.0125(21)	5.08(26)	0.629(8)	190(20)	1.314(10)	—	—
Cd(NO_3_)_2_	0.0089(3)	1.387(16)	0.6115(19)	53(2)	1.2504(21)	—	—
Cd(ClO_4_)_2_	0.000886(7)	0.5475(14)	0.6692(11)	178(3)	1.1321(17)	—	—
CdSO_4_	0.0127(7)	3.69(5)	0.522(11)	67(7)	1.062(24)	—	—
CaBr_2_	0.00217(8)	0.788(14)	0.635(6)	90(5)	1.116(13)	—	—
CaCl_2_	0.000328(25)	0.364(17)	0.830(16)	1100(200)	1.282(17)	1600(200)	2.654(15)
CaI_2_	0.00160(5)	0.697(7)	0.647(4)	112(6)	1.125(7)	—	—
Ca(NO_3_)_2_	0.027(3)	1.87(4)	0.539(12)	17(3)	1.16(3)	—	—
Ca(ClO_4_)_2_	0.00166(6)	0.713(9)	0.645(6)	111(7)	1.129(11)	—	—
CsAc	0.03935(7)	0.7497(20)	0.5874(29)	—	—	—	—
CsBrO_3_	1	3.35(3)	0.5796(23)	—	—	—	—
CsBr	0.463(5)	2.1458(20)	0.5738(14)	—	—	—	—
CsClO_3_	1	3.37(4)	0.5806(26)	—	—	—	—
CsCl	0.398(5)	2.0339(18)	0.5742(17)	1000(1000)	6.8(9)	—	—
CsF	0.06227(15)	0.9032(14)	0.5902(16)	—	—	—	—
CsOH	0.0468(5)	0.7673(19)	0.5792(31)	—	—	—	—
CsI	1	2.582(8)	0.5438(13)	—	—	—	—
CsNO_3_	1	4.61(9)	0.655(7)	—	—	—	—
CsClO_4_	1	4.12(6)	0.602(3)	—	—	—	—
Cs_2_SO_4_	0.0145(22)	1.90(10)	0.627(5)	63(6)	1.301(12)	—	—
ChBr	0.540(7)	3.134(3)	0.6140(19)	—	—	—	—
ChCl	0.2243(14)	2.1110(20)	0.6269(19)	—	—	—	—
CrCl_3_	0.03002(21)	3.358(8)	0.557(5)	—	—	—	—
Cr(NO_3_)_3_	0.03592(31)	3.504(9)	0.535(6)	—	—	—	—
Cr_2_(SO_4_)_3_	0.080(5)	10.85(4)	0.508(13)	—	—	—	—
CoBr_2_	0.02628(16)	1.904(16)	0.609(4)	—	—	—	—
CoCl_2_	0.03964(10)	2.126(8)	0.5664(22)	—	—	—	—
CoI_2_	0.02031(23)	1.902(32)	0.632(6)	—	—	—	—
Co(NO_3_)_2_	0.00772(27)	1.252(16)	0.598(5)	36(3)	1.131(14)	—	—
Co(ClO_4_)_2_	0.001004(18)	0.555(5)	0.6455(19)	123(2)	1.092(4)	—	—
CoSO_4_	1	9.97(12)	0.454(4)	—	—	—	—
CuBr_2_	0.0334(6)	1.926(4)	0.5565(27)	130(20)	2.56(9)	—	—
CuCl_2_	0.050(10)	2.34(7)	0.548(16)	27(1)	1.81(16)	—	—
Cu(NO_3_)_2_	0.0130(9)	1.429(31)	0.574(7)	21(3)	1.097(27)	—	—
Cu(ClO_4_)_2_	0.001184(13)	0.595(3)	0.6439(17)	114(2)	1.097(3)	—	—
CuSO_4_	0.468(30)	9.35(4)	0.451(5)	—	—	—	—
Gdn_2_CO_3_	1	8.388(17)	0.6014(9)	—	—	—	—
GdnBr	1	2.38(6)	0.531(6)	2.83(3)	1.220(21)	—	—
GdnCl	1	1.84(12)	0.486(12)	2.92(10)	1.002(22)	—	—
GdnI	1	1.86(8)	0.486(9)	3.08(5)	1.136(22)	—	—
GdnNO_3_	1	3.34(9)	0.583(6)	15(4)	1.80(11)	—	—
GdnClO_4_	1	2.1(8)	0.51(6)	4.58(23)	1.09(16)	—	—
HBr	0.00272(4)	0.2921(27)	0.6857(24)	37.7(7)	1.190(6)	—	—
HCl	0.009(8)	0.36(24)	0.66(4)	7(1)	1.03(21)	—	—
HF	0.011(4)	5.5(4)	0.522(19)	140(40)	1.189(16)	—	—
HI	0.02000(8)	0.5701(7)	0.6002(15)	−90(10)	2.93(7)	—	—
HNO_3_	0.07324(17)	0.8772(24)	0.5546(26)	—	—	—	—
HClO_4_	0.0381(4)	0.7332(7)	0.5889(19)	−52(2)	2.527(26)	—	—
FeCl_2_	0.04413(20)	2.271(5)	0.5601(17)	—	—	—	—
k_2_succ	0.195(19)	2.583(15)	0.468(9)	—	—	—	—
LiAc	0.08644(31)	1.0147(19)	0.5774(20)	—	—	—	—
LiBr	0.0363(9)	0.788(6)	0.617(9)	800(300)	6.1(4)	−60(10)	2.90(15)
LiClO_3_	0.0333(6)	1.07(12)	0.374(30)	—	—	—	—
LiCl	0.00261(14)	0.325(6)	0.753(11)	86(9)	1.319(14)	164(9)	3.61(6)
LiTFSI	0.0026	0.32	0.75	76(4)	1.27(3)	129(2)	3.1(1)
LiF	1	2.362(25)	0.523(4)	—	—	—	—

**Table 2:** Fit parameters for ln *γ*, part 2

Salt	*x*_h,Dipole_	*D*_Dipole_	*λ*_Dipole_	*D*_Quadrupole_	*λ*_Quadrupole_	*D*_Octupole_	*λ*_Octupole_
LiOH	0.014(5)	0.78(10)	0.73(4)	40(20)	1.42(4)	—	—
LiI	0.001457(28)	0.2372(17)	0.729(4)	86(3)	1.254(6)	—	—
LiNO_3_	0.0353(10)	0.785(6)	0.6099(14)	3.93(26)	1.393(5)	—	—
LiClO_4_	0.00438(18)	0.316(8)	0.631(5)	14.7(8)	1.091(17)	—	—
Li_2_SO_4_	0.197(5)	3.536(9)	0.523(5)	—	—	—	—
MgBr_2_	0.00197(9)	0.742(18)	0.625(5)	79(3)	1.086(12)	—	—
MgCl_2_	0.00197(29)	0.74(5)	0.613(14)	75(7)	1.07(3)	—	—
MgI_2_	0.00144(17)	0.611(25)	0.647(11)	98(4)	1.096(22)	—	—
Mg(NO_3_)_2_	0.0034(8)	0.89(9)	0.544(20)	34(2)	0.98(5)	—	—
Mg(ClO_4_)_2_	0.01902(6)	1.510(5)	0.5686(23)	—	—	—	—
MgSO_4_	0.0060(12)	3.04(14)	0.572(18)	130(30)	1.120(29)	—	—
na_2_succ	0.0700(14)	2.144(5)	0.500(4)	—	—	—	—
KAc	0.04351(8)	0.7879(20)	0.5904(23)	—	—	—	—
KBrO_3_	1	3.49(8)	0.599(6)	—	—	—	—
KBr	0.2433(28)	1.4285(21)	0.5513(21)	—	—	—	—
KClO_3_	1	3.67(12)	0.624(10)	—	—	—	—
KCl	0.2797(31)	1.5296(14)	0.5557(21)	—	—	—	—
KF	0.0152(4)	0.605(4)	0.6420(23)	14.8(6)	1.235(6)	—	—
K_2_HPO_4_	0.45(6)	4.78(12)	0.518(6)	—	—	—	—
KH_2_PO_4_	1	4.62(8)	0.655(7)	—	—	—	—
KOH	0.0476(5)	0.945(18)	0.714(8)	—	—	—	—
KI	0.1878(19)	1.2326(20)	0.5417(21)	—	—	—	—
KNO_3_	1	2.92(7)	0.564(4)	5.28(15)	1.336(32)	—	—
KClO_4_	1	3.37(4)	0.5806(26)	—	—	—	—
K_2_SO_4_	1	6.18(10)	0.498(5)	—	—	—	—
KSCN	0.606(23)	1.739(4)	0.514(4)	—	—	—	—
RbAc	0.04085(5)	0.7767(13)	0.5922(14)	—	—	—	—
RbBrO_3_	1	3.02(4)	0.5672(30)	—	—	—	—
RbBr	0.441(11)	1.809(4)	0.5446(29)	—	—	—	—
RbClO_3_	1	2.91(31)	0.547(26)	—	—	—	—
RbCl	0.354(5)	1.7027(30)	0.5507(29)	—	—	—	—
RbF	0.0207(26)	0.717(27)	0.647(7)	18(2)	1.458(19)	—	—
RbI	0.402(7)	1.8067(26)	0.5518(23)	—	—	—	—
RbNO_3_	1	2.98(5)	0.5676(31)	4.65(4)	1.247(17)	—	—
RbClO_4_	1	3.883(28)	0.5968(17)	—	—	—	—
Rb_2_SO_4_	1	5.365(11)	0.4824(10)	—	—	—	—
AgNO_3_	1	2.93(5)	0.564(4)	5.20(3)	1.265(10)	—	—
NaAc	0.05354(13)	0.8300(13)	0.5769(19)	—	—	—	—
NaBrO_3_	1	2.863(24)	0.580(4)	—	—	—	—
NaBr	0.019(4)	0.54(8)	0.590(16)	5.3(5)	1.02(8)	—	—
NaClO_3_	1	2.015(8)	0.5114(17)	—	—	—	—
NaCl	0.0132(4)	0.548(7)	0.631(5)	14.5(9)	1.208(13)	—	—
NaF	0.49(4)	1.89(3)	0.551(5)	—	—	—	—
NaFo	0.1622(27)	1.1535(25)	0.547(4)	—	—	—	—
NaHCO_3_	1	2.602(21)	0.5446(28)	—	—	—	—
NaH_2_PO_4_	1	3.985(29)	0.644(4)	—	—	—	—
Na_2_HPO_4_	1	6.56(7)	0.537(4)	—	—	—	—
NaOH	0.00609(29)	0.476(8)	0.724(12)	50(6)	1.347(20)	240(30)	5.04(21)
NaI	0.00851(27)	0.472(5)	0.651(4)	19(1)	1.224(11)	3000(3000)	7.1(6)
NaNO_3_	0.000160(12)	0.0763(27)	0.859(12)	560(50)	1.261(15)	710(60)	2.321(26)
NaClO_4_	0.0358(16)	0.794(9)	0.6119(25)	8.9(4)	1.307(5)	—	—
Na_2_SO_4_	1	5.937(28)	0.5004(19)	—	—	—	—
NaSCN	0.0780(8)	0.957(7)	0.587(9)	—	—	—	—
SrBr_2_	0.001878(17)	0.7420(20)	0.6433(10)	108(1)	1.1316(15)	—	—
SrCl_2_	0.00382(4)	1.0243(29)	0.6354(10)	80(1)	1.1635(20)	—	—
SrI_2_	0.001399(10)	0.6480(14)	0.6468(8)	119(1)	1.1206(12)	—	—
Sr(NO_3_)_2_	0.038(9)	1.93(7)	0.503(19)	15(4)	1.10(4)	—	—
Sr(ClO_4_)_2_	0.0065(5)	1.15(3)	0.597(8)	30(5)	1.083(31)	—	—
ZnBr_2_	0.00339(25)	1.021(22)	0.678(9)	160(20)	1.323(7)	—	—
ZnCl_2_	0.00179(5)	0.830(7)	0.672(4)	191(7)	1.212(5)	243(9)	3.047(8)
ZnF_2_	0.148(8)	4.89(7)	0.5924(19)	—	—	—	—
ZnI_2_	0.0050(11)	1.10(7)	0.672(24)	130(30)	1.429(22)	—	—
Zn(NO_3_)_2_	0.0084(4)	1.18(3)	0.575(7)	23(2)	1.055(27)	—	—
Zn(ClO_4_)_2_	0.00094(13)	0.50(3)	0.609(8)	78(3)	1.004(19)	—	—
ZnSO_4_	1	10.126(24)	0.4191(9)	−400(60)	3.05(6)	—	—

**Fig. 2:**
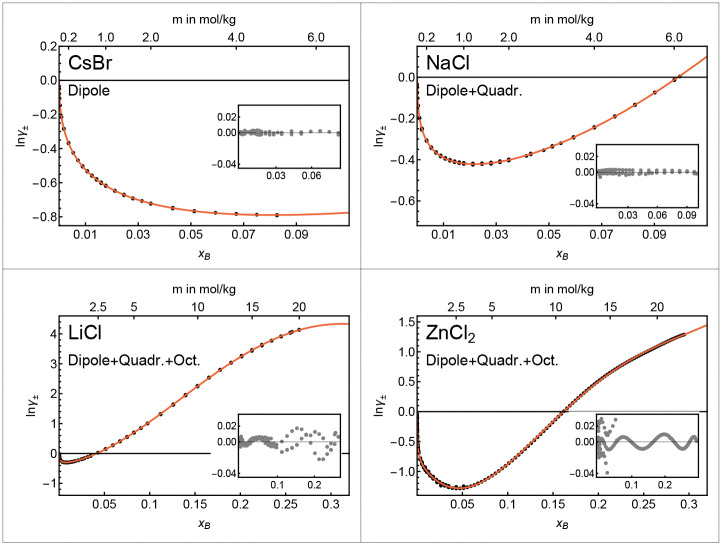
Example fits of ln *γ*_±_ for different electrolytes using the model description from eqn (4). The insets show the fit residuals.

Fig. 2 shows example fits of individual electrolytes. For CsBr, which does not display strong cation–anion interactions, the dipole expansion represents the activity coefficient sufficiently well over the full data range. When increasing the cation–anion interaction strength, such as for the cases of LiCl and ZnCl_2_, complex ion–ion correlations occur in the solution.

In previous molecular dynamics and X-ray studies, these were shown to result in spatial inhomogeneities due to ion clustering.^21–23^ In our framework, the effect of these spatial inhomogeneities is encoded in the charge distributions within and outside the probe volume. With increasing ion clustering, the charge distributions become more complex, resulting in larger contributions from quadrupolar and octupolar terms in our model (see eqn (4)). Accordingly, the quadrupole and octupole terms of our statistical model capture the more complex behavior of LiCl and ZnCl_2_ solutions over the whole mole fraction range. The number of contributing multipole components provides a quantification of the impact of (multi-body) ion–ion correlations: complex ion–ion correlations require stronger contributions from an increased number of higher-order multipole expansion terms.

## 3 Discussion

**Fig. 3:**
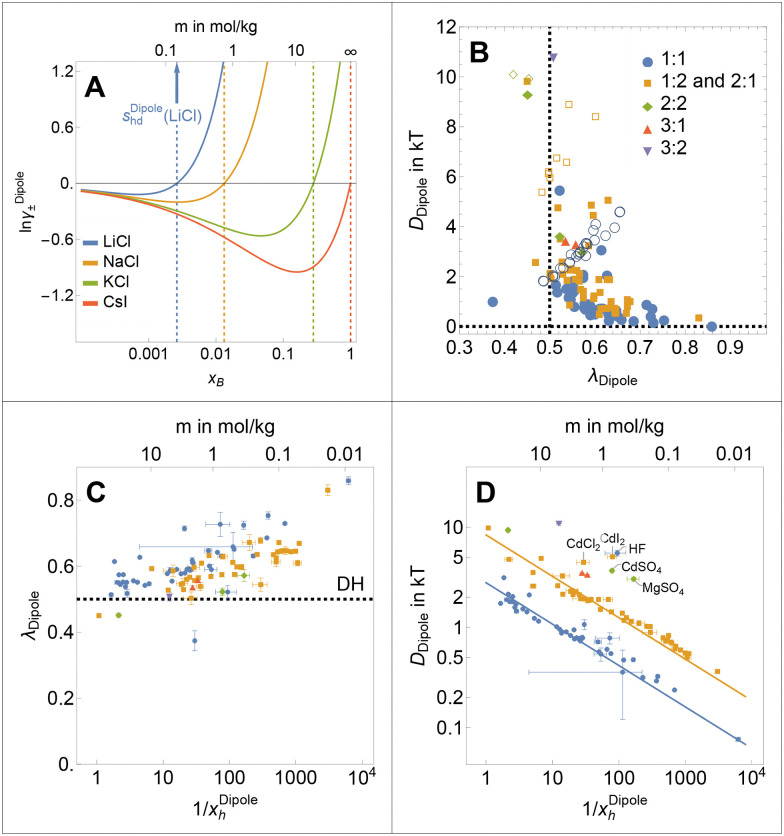
Comparison of different electrolyte solutions. (A) Examples of the dipolar part of ln *γ*_±_ for different electrolytes. The crossover from negative to positive values marks the transition from dipole–dipole to more complex interaction forms. 1/*x*_B_ corresponds to the size of the probe volume. (B) Depth of the dipolar part of the potential *versus* exponent. Electrolytes with *x*^Dipole^_h_ = 1 are shown with open markers. (C) Exponent *λ*_Dipole_*versus* 1/*x*^Dipole^_h_, of the hydrated electrolyte. The dashed line represents the coefficient expected for the equivalent Debye–Hückel limiting law. (D) Amplitude *D*_Dipole_ for electrolytes with *x*^Dipole^_h_ < 1. The data were fitted with a power law of the form 
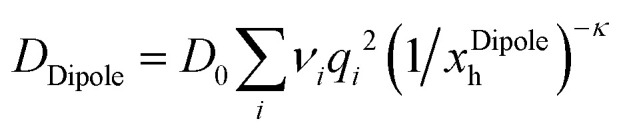
 with *D*_0_ = 2.81(8) and *κ* = 0.42(1). The lines display the power law for 1 : 1 (blue) and 2 : 1 and 1 : 2 (yellow) electrolytes. A few electrolytes with exceptional properties are labeled separately.

Summarizing the results above, we have demonstrated that a multipole expansion of the interaction between the central observation volume and the surrounding solution is suitable to represent ln *γ*_±_ (see eqn (4)). Each expansion exponent, *λ*_*l*_, reflects the combination of the thermally averaged interaction between the multipole inside the observation volume and the charge distribution in its environment (Fig. 3). The interaction spatially decays as 
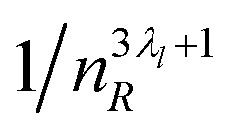
. The dipole–dipole interaction term (*l* = 1 in eqn (4)) compares well with the simplest form of the Debye–Hückel law (ln *γ*_±_ ∝ *m*_B_^1/2^) for dilute solutions where *x* ∝ *m*_B_ when choosing *λ*_Dipole_ = 0.5 (see Fig. 3C).

Fig. 3 shows the results of the dipolar contribution for 135 electrolytes. (See fit parameters in Tables 1 and 2 and the classification of the investigated electrolytes in Table 3 in the Appendix.) In panel (A), we show the dipolar contribution to ln *γ*_±_ for four electrolytes with increasing interaction strength from CsI to LiCl. The crossover point *x*^Dipole^_h_ (dashed vertical line) marks a boundary: dipole–dipole interaction becomes unfavorable, *i.e.*, endothermic, for higher concentrations, and complex (*i.e.*, quadrupole and octupole) interactions become more important. The radius of the observation volume where this happens is determined by 1/*x*^Dipole^_h_. With increasing ion–ion and ion–water interactions, the crossover shifts toward lower concentrations. This is in qualitative agreement with findings from a combined SAXS and simulation study (Fig. 3 in (ref. 22)) showing that a crossover from Debye–Hückel to more complex behavior occurs at lower concentrations for more strongly interacting ions.

**Table 3:** Electrolytes classified according to Fig. 3

Electrolyte class	Members
1 : 1, *x*_h_ < 1	ChBr, ChCl, CsAc, CsBr, CsCl, CsF, CsOH, HBr, HClO_4_, HF, HI, HNO_3_, KAc, KBr, KCl, KF, KI, KOH, KSCN, LiAc, LiBr, LiCl, LiClO_3_, LiClO_4_, LiI, LiNO_3_, LiOH, NaAc, NaBr, NaCl, NaClO_4_, NaF, NaFo, NaI, NaNO_3_, NaOH, NaSCN, NH_4_Br, NH_4_Cl, RbAc, RbBr, RbCl, RbF, RbI
1 : 1, *x*_h_ = 1	AgNO_3_, CsBrO_3_, CsClO_3_, CsClO_4_, CsI, CsNO_3_, GdnBr, GdnCl, GdnClO_4_, GdnI, GdnNO_3_, HCl, KBrO_3_, KClO_3_, KClO_4_, KH_2_PO_4_, KNO_3_, LiF, NaBrO_3_, NaClO_3_, NaH_2_PO_4_, NaHCO_3_, NH_4_ClO_4_, NH_4_NO_3_, NH_4_SCN, RbBrO_3_, RbClO_3_, RbClO_4_, RbNO_3_
2 : 1 and 1 : 2, *x*_h_ < 1	BaBr_2_, BaCl_2_, BaOH_2_, BaI_2_, Ba(ClO_4_)_2_, CdBr_2_, CdCl_2_, CdI_2_, Cd(NO_3_)_2_, Cd(ClO_4_)_2_, CaBr_2_, CaCl_2_, CaI_2_, Ca(NO_3_)_2_, Ca(ClO_4_)_2_, CoBr_2_, CoCl_2_, CoI_2_, Co(NO_3_)_2_, Co(ClO_4_)_2_, CuBr_2_, CuCl_2_, Cu(NO_3_)_2_, Cu(ClO_4_)_2_, FeCl_2_, K_2_HPO_4_, MgBr_2_, MgCl_2_, MgI_2_, Mg(NO_3_)_2_, Mg(ClO_4_)_2_, SrBr_2_, SrCl_2_, SrI_2_, Sr(NO_3_)_2_, Sr(ClO_4_)_2_, ZnBr_2_, ZnCl_2_, ZnF_2_, ZnI_2_, Zn(NO_3_)_2_, Zn(ClO_4_)_2_
2 : 1 and 1 : 2, *x*_h_ = 1	Ba(NO_3_)_2_, Gdn_2_CO_3_, K_2_SO_4_, Na_2_HPO_4_, Na_2_SO_4_, (NH_4_)_2_HPO_4_, (NH_4_)_2_SO_4_, Rb_2_SO_4_
2 : 2, *x*_h_ < 1	CdSO_4_, CuSO_4_, MgSO_4_, ZnSO_4_
2 : 2, *x*_h_ = 1	CoSO_4_
3 : 1, *x*_h_ < 1	CrCl_3_, Cr(NO_3_)_3_
3 : 2, *x*_h_ < 1	Cr_2_(SO_4_)_3_)

Panel (B) shows the depth of the dipolar contribution to ln *γ*_±_*versus* its exponent for the different 1 : 1 to 3 : 2 electrolytes. We display electrolytes with *x*^Dipole^_h_ = 1, *i.e.*, where no crossover is observed, with open markers. This class contains both electrolytes that are well-described by the dipolar term, only, as well as special cases such as many guanidinium and sulfate salts for which quadrupolar contributions are important, despite no crossover being observed in the dipolar term. The vertical line at *λ*_Dipole_ = 0.5 marks the value corresponding to the Debye–Hückel (DH) law. Electrolytes with large *D*_Dipole_ typically show exponents close to the DH-value. A comparison to panels (A) and (C) shows that *D*_Dipole_ is determined by both the strength of the electrolyte and the limiting concentration, where higher-order terms become more prominent. A larger exponent indicates an increased importance of thermal averaging effects (remember that thermal averaging changes the 1/*R*^3^-interaction of close dipoles to a 1/*R*^6^-dependency, when the effective interaction strength is much smaller than *kT*).

Panel (C) supports this picture. Here, we observe that (a) electrolytes with smaller 1/*x*^Dipole^_h_ and (b) electrolytes with higher charge show exponents closer to the DH value. Electrolytes where higher order contributions dominate at low concentrations (*i.e.*, *x*^Dipole^_h_ is small) or which only weakly interact with each other and with water (*e.g.*, 1 : 1, blue, *vs.* 2 : 1 and 1 : 2, yellow) tend to have larger exponents.

Panel (D) in Fig. 3 shows the interaction strength, *D*_Dipole_, as function of 1/*x*_h_ for 97 electrolytes. We observe a power law of the form *D*_Dipole_ = *D*_0_*I*_*m*_(1/*x*^Dipole^_h_)^−*κ*^ with *D*_0_ = 2.81(8) and *κ* = 0.42(1), where 
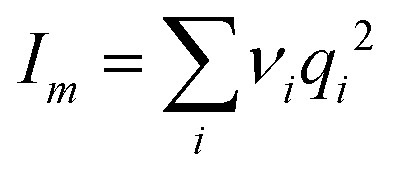
 is the ionic strength of the electrolyte in our molecular frame. The proportionality of the interaction strength to *I*_*m*_ is in agreement with the ionic strength dependency of the interaction energy as described by Debye and Hückel (see Appendix for a detailed comparison). We noticed a few exceptional cases which are, however, beyond the scope of our discussion.

Another interesting property of eqn (3) resulting from our model is that the weighting function (eqn (3)) is directly related to the microscopic radius-dependent partition function, *Z*_*l*_. The weighting function originates from the excess charge, Δ*q*(*n*_*R*_), generated by the potential of the central observation volume at the given position. By assuming a Van’t Hoff-like equation for each multipolar expansion term, we can relate each Δ*q*_*l*_(*n*_*R*_) to a microscopic osmotic pressure *Π*_*l*_(*n*_*R*_)6
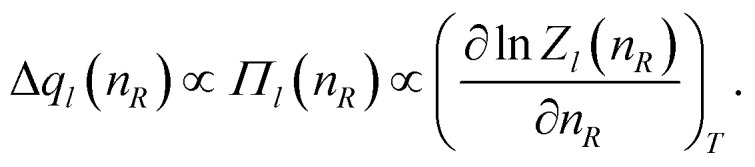


Since the angular dependency of the charge difference is described by spherical harmonics, this radius dependency describes the changes along the major axes (*e.g.*, along the dipole axis, for dipole–dipole interaction, see Fig. 1B). Integration of eqn (3) including the pre-factor *U*_*l*_ yields7
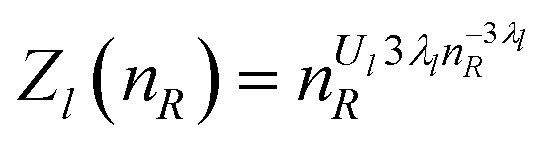
so that *λ*_*l*_ and *U*_*l*_ determine the general shape and the sharpness of the partition function, respectively.

**Fig. 4:**
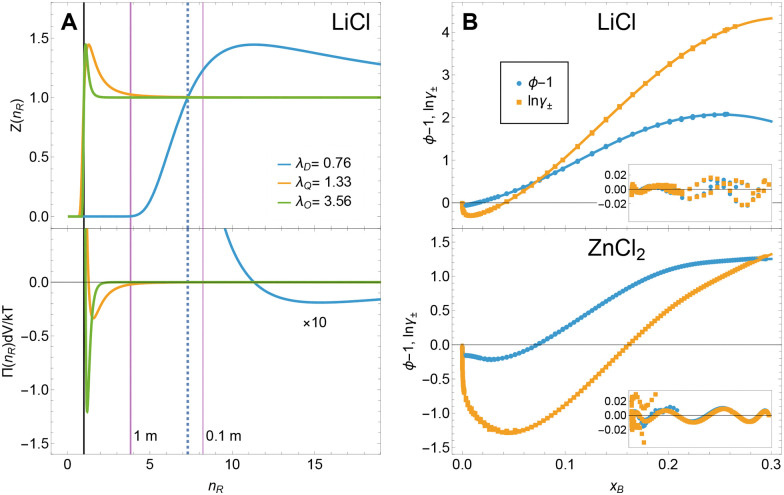
(A) Microscopic insights from our new description: radius dependency of the contributions to the partition function (top) and the osmotic pressure (bottom) for LiCl. For clarity, we used the potential depth *U*_*l*_ = 1. The long-range dipole interaction shows a crossover from positive to negative values at *n*^Dipole^_*R*_ = 7.3 (dashed blue) where *n*^Dipole^_*R*_ = (1/*x*^Dipole^_h_)^1/3^. Purple: *n*_*R*_ values for 1 m and 0.1 m solutions. (B) Example global fits of the osmotic and activity coefficients for LiCl (top) and ZnCl_2_ (bottom) using eqn (4) and (8). The insets show the fit residuals.

Fig. 4A, top shows the contributions to the partition function for LiCl using *U*_*l*_ = 1 for better comparability. The long-range interaction shows a gradual transition when hitting the crossover (dashed blue), where dipole–dipole interaction is replaced by higher-order, more complex interactions. In contrast, the quadrupole (yellow) and octupole (green) terms show a sharp decay at *n*_*R*_ = 1.

The corresponding osmotic pressures are shown in the bottom part of Fig. 4A. The dipole contribution is weak and only attractive (*i.e.*, negative) at long distances. In contrast, the quadrupole and octupole terms are short-range and still attractive at the highest possible concentrations.

In addition, the description of ln *γ*_±_ in eqn (4) allows us to derive an analytical form of the osmotic coefficient. The Gibbs–Duhem equation^27^ allows to convert ln *γ*_±_ to the osmotic coefficient8
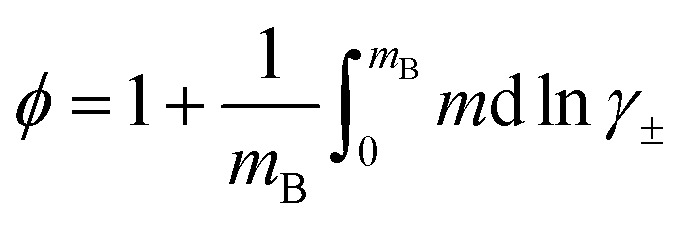
which is closely related to the water activity9
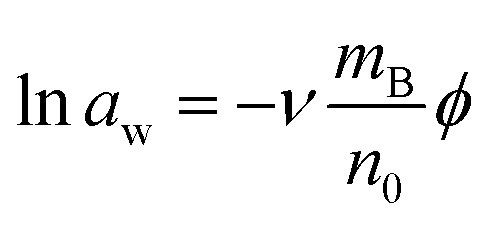
with *n*_0_ as moles of water in 1 kg of the solvent. When we describe molality as *m*_B_ = *n*_0_*x*_B_/(1 − *x*_B_) and use eqn (4) to integrate eqn (8) we obtain10
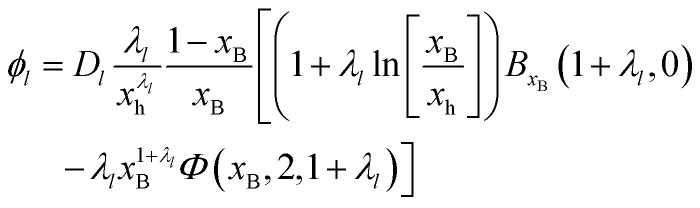
for the *l*th order contribution to the osmotic coefficient. Here, *B*_*x*_B__(1 + *λ*_*l*_, 0) is the incomplete Euler Beta function^28^ and *Φ*(*x*_B_, 2, 1 + *λ*_*l*_) is the Lerch transcendent^29^ (see Appendix for details on the integration procedure and the special functions).

These analytical descriptions (eqn (4) and (8)) allow a direct global fit of experimental values of electrolyte activity, osmotic coefficient, and water activity and, therefore, simplify the retrieval of excess thermodynamic properties using different measurement techniques. Fig. 4B shows example fits for LiCl and ZnCl_2_ together with their fit residuals (insets). Please note that the oscillatory behavior in the residuals is an artifact, since the published data have been fitted by a combination of Debye–Hückel, Pitzer, and polynomial terms. In case of ZnCl_2_ Goldberg^30^ required 13 and 8 coefficients to reproduce ln *γ*_±_ and the osmotic coefficient, respectively, while we need a total of seven identical fit parameters with a clear physical meaning (*σ*_ln*γ*_±__ = 0.00983 and *σ*_*ϕ*_ = 0.00589 *vs. σ*_ln*γ*_±__ = 0.00747 and *σ*_*ϕ*_ = 0.00684 in the work by Goldberg) for both physical properties. We have published the data sets used for the fit as well as the fit parameters and predicted values for ln *γ*_±_, *ϕ*, and *a*_w_ separately in machine-readable form.^31^

**Fig. 5:**
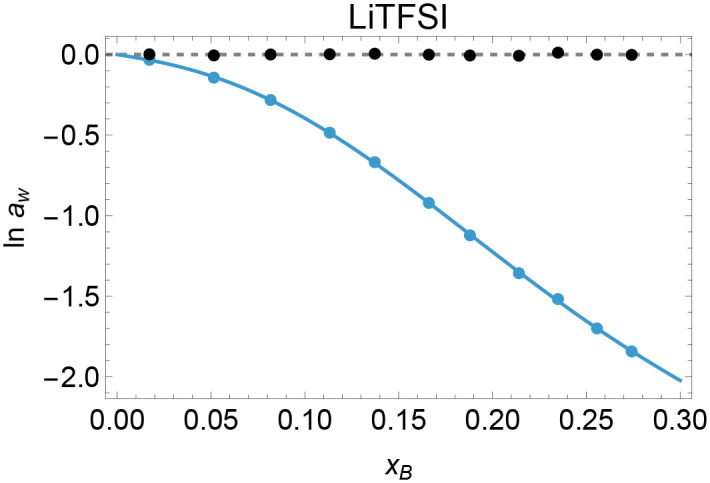
Blue points: water activity data, ln *a*_w_ (Zhigalenok *et al.*^32^) of the “water in salt” electrolyte LiTFSI as function of electrolyte mole fraction. Blue line: fit of ln *a*_w_ based on our multipole expansion, which requires dipole plus quadrupole plus octupole terms. Due to the lack of low-concentration data, we used the LiCl parameter for the dipolar term to obtain a stable fit. Black dots: fit residuals.

Finally, we tested if the generalized multipole expansion approach applies to “water in salt (WISE)” electrolytes such as lithium-bis-(trifluoromethanesulfonyl)-imide (LiTFSI), see Fig. 5. Water activity data for this compound were reported recently by Zhigalenok *et al.*^32^ for intermediate to high LiTFSI concentrations. Due to a lack of low-concentration data, we fixed the dipolar set of parameters to those of LiCl to obtain a numerically stable fit. As shown by the residuals (black dots) in Fig. 5, our model is well suited to represent the water activity of LiTFSI over the full concentration range. As expected for systems with strongly inhomogeneous charge distributions,^2^ we must include the dipole plus quadrupole plus octupole terms.

## 4 Conclusions

We have demonstrated above a novel statistical approach to model and understand the excess thermodynamic functions, *i.e.*, (ln *γ*_±_, osmotic coefficient, *ϕ*, and water activity, *a*_w_) of aqueous electrolyte solutions and applied it to 135 electrolytes. The foundation of our coarse-grained approach is a generalized multipole expansion which describes the interaction of all charges outside a purely statistical, stoichiometrically defined observation volume with the potential originating from the charge distribution inside. A key feature is that charge distributions inside and outside the observation volume depend both on ions and water. This allows us to take into account the properties of the hydration water network as a function of concentration beyond continuum model descriptions that reduce water's influence to the dielectric constant.

**Fig. 6:**
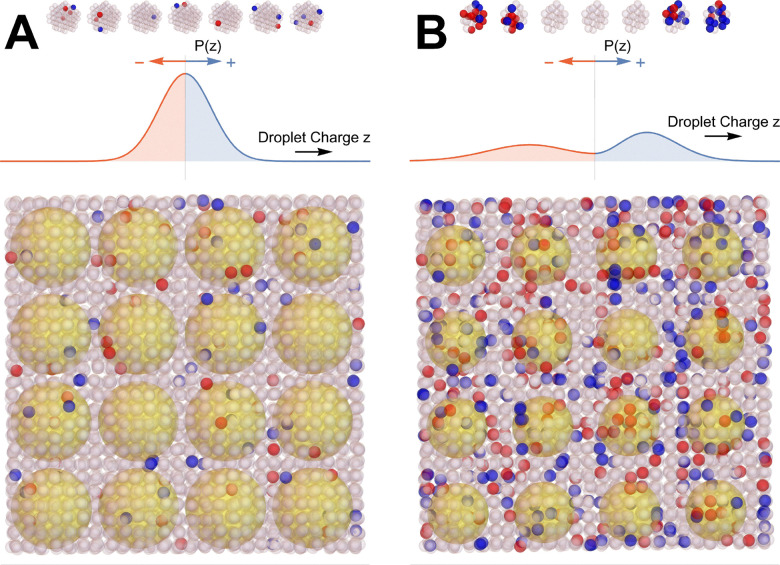
Our simplifying statistical approach efficiently describes the thermodynamic properties of complex electrolyte solutions: (A) Weakly interacting ions or dilute solutions (*e.g.*, CsBr, see Fig. 2) show a more homogeneous charge distribution (anions in red, cations in blue). Top: Illustration of typical instantaneous charge distributions within the observation volume. Center: Qualitative probability distribution of the instantaneous observation volume net charge. Regardless of the distribution, the average observation volume charge is zero by construction. Bottom: Illustration of instantaneous ion distributions inside and outside a selection of observation volumes (yellow spheres). The dipole term in our model is sufficient to describe the thermodynamics of these cases. (B) Same set of illustrations for complex electrolytes at high concentrations where ion clustering starts to become important (*e.g.*, LiCl, ZnCl_2_, see Fig. 2). Even for these cases, the quadrupole and octupole terms in our model fully capture the effect of the increasing structural heterogeneity on the thermodynamics.

We summarize the key features of our description in Fig. 6: with increasing concentration, the charge distribution within our observation volume captures the emergence of spatial inhomogeneities, such as ion clustering. This becomes more complex with increasing concentration from panel (A) to (B), while still obeying, on average, the constraints of being charge-neutral by containing one electrolyte unit and the stoichiometric amount of water. The increased complexity of the charge distribution results in larger higher-order contributions to our multipole expansion.

Even for the most complex studied electrolyte solutions, the 3rd-order expansion, including dipole, quadrupole, and octupole contributions, is found sufficient to describe the full set of thermodynamic properties in the whole solubility range. This is a substantial simplification compared to existing heuristic descriptions.

In addition, as summarized in Fig. 6, our statistical perspective allows us to collapse the effect of the complex structural heterogeneity found in experimental data and simulations for strongly interacting ions and concentrated solutions into the quadrupolar and octupolar terms of our thermodynamic functions. For example, recent studies suggest that a Kirkwood transition, *i.e.*, the concentration at which the Debye length is no longer the characteristic length scale in an electrolyte solution, happens at lower concentrations for strongly interacting electrolytes due to ion clustering.^22,23^ This observation is in qualitative agreement with our findings in Fig. 2 and 8A, where quadrupole and octupole terms start to contribute significantly at lower concentrations for strongly interacting electrolytes.

Our universal approach is not restricted to binary electrolyte solutions but is generalizable to electrolyte mixtures and solutions in general. This process requires an additional summation over all possible (neutral) solute combinations in the observation volume and their interaction with the solute mixture in the environment. The change of perspective, we propose, offers new insights into the average thermodynamic properties of electrolyte solutions and their connection to local structural heterogeneity and ion complexes as they are characterized by state-of-the-art spectroscopic and simulation techniques. We hope these findings will contribute to the understanding of concentrated electrolyte solutions, which are relevant in many biological and electrochemical applications in today's society.

## Author contributions

G. S.: conceptualization, data curation, formal analysis, investigation, methodology, software, visualization, writing – original draft, writing – review & editing. S. P.: conceptualization, writing – original draft, writing – review & editing.

## Conflicts of interest

There are no conflicts of interest to declare.

## Figures and Tables

**Fig. 7:**
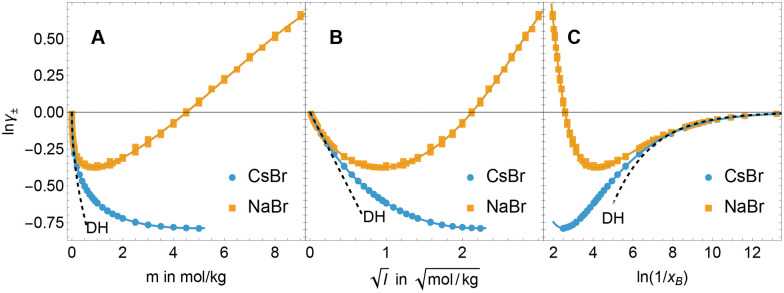
Logarithm of the activity coefficient (ln *γ*_±_) for cesium bromide and sodium bromide in different coordinate systems: (A) molality, (B) ionic strength, and (C) *r*_B_ = ln(1/*x*_B_). The dashed curve shows the Debye–Hückel limiting law for a 1 : 1 electrolyte in the different coordinate systems.

## Data Availability

Data for this article, including the activity data used for the model fitting, the fit parameters as well as the resulting recommended activity coefficients, osmotic coefficients and water actvities are available at TUDOData (RESOLV-data) at https://doi.org/10.17877/RESOLV-2024-M4WGMZTG, 2025.

## Appendices

### Appendices

#### Heuristic discovery of eqn (3) and (4)

Fig. 7 shows ln *γ*_±_ for cesium bromide and sodium bromide as a function of molality (A), ionic strength (B), and the log-transformed coordinate *r*_B_ = ln(1/*x*_B_) = ln(1 + *N*_w_) (C). As defined above, *N*_w_ is the number of water molecules per electrolyte unit (*i.e.*, per *ν*_−_ anions and *ν*_+_ cations). While ln *γ*_±_ shows the well-known complex mol fraction and ionic strength dependency which is typically described by Debye–Hückel or Pitzer parameters, in the log-transformed coordinate system (panel (C)) ln *γ*_±_ disappears for *r*_B_ → ∞ and shows a curve similar to a Morse potential. Indeed, in this coordinate system ln *γ*_±_ can be well represented over the full concentration range when using a small number (*l* ≤ 3) of fit functions of the form

11

A back-transform to mol fraction units recovers eqn (4).

This simple functional form led us to the conclusion that a (spherical) charge-neutral observation volume containing *ν*_−_ anions, *ν*_+_ cations, and *N*_w_ water molecules has a special meaning because it represents the statistical average over all possible configurations of that size. In addition, the question arose how the dipole and higher multipole moments of this observation droplet interact with the (again statistically averaged) excess charge distributions in the environment (eqn (2) and (21)). After removal of the angular dependencies, we chose the weight function such that the full integral recovered eqn (3).

#### Definitions of distances

We have introduced the hydrated electrolyte radius, *R*_h_, in the main text without giving further specifications. We define this radius from density measurements^26^ and the composition of the solution as

12

where *N*_h_ is the hydration shell size (*i.e.*, the number of water molecules per *ν*_+_ cations and *ν*_−_ anions) where the solvated ion complexes start to repel each other, and *N*_A_ is Avogadro's constant. In cases where we could not obtain *N*^dipole^_h_ from the activity data, we fixed its value to *N*^dipole^_h_ = 0, see Tables 1–3. This value corresponds to a (hypothetical) contact ion pair without a solvation shell around the ions as a reference unit and yields *x*_h_ = 1. In general, *N*_h_ will be different for different orders of the multipole expansion. Heuristically, we found that we could represent the electrolyte activities well when allowing *N*^Dipole^_h_ ≥ 0 (this value corresponds to *x*^Dipole^_h_ ≤ 1) while keeping *N*_h_ = 0 (*i.e.*, *x*_h_ = 1) for the quadrupole and octupole contributions. Please note that *x*_h_ is a fit parameter which results from a macroscopic average over a distribution of microscopic states. We have neither information on the higher moments of this distribution nor the number of contact ion pairs in the solution. Supplementary techniques will have to provide this information.

The average apparent molar volume in the solution is given by *φ̄*_sol_ = *M̄*_sol_/*ρ*_sol_ where *M̄*_sol_ = *xM*_B_ + (1 − *x*)*M*_w_ and *ρ*_sol_ are the average molar mass and the density of the solution, respectively.

Similarly, the radius of the “observation volume” is defined by

13

where *N*_w_ = *x*_w_/*x*_B_ is the number of water molecules per electrolyte unit.

#### Spherical harmonics and special functions

##### Spherical harmonics and their application to electrolyte solutions

According to Chapter 5, Volume 2 (The classical theory of fields) in the Landau–Lifshitz series of textbooks^33^ the action of a field of a set of charges inside our spherical probe volume with radius *R*_d_ on a charge *q* at a distance *R*_*q*_ > *R*_d_ from the center of the probe volume can be developed as multipole expansion

14

where the *l*th term of the potential is given by

15

Here, *l* ≥ 0 and *m* are integer numbers, and *Θ* and *Φ* are the angles that describe the direction of *R*_*q*_ with respect to the coordinate system. The *z*-axis of the coordinate system is typically defined by the dipole moment of the distribution. The multipole moments

16

originate from the distribution of the set of charges, *e*_*a*_ at the distances *r*_*a*_ ≤ *R*_d_ and the angles *θ*_*a*_ and *ϕ*_*a*_ inside our probe volume. For simplicity, we have used point charges. However, this approach is easily transferable to systems described by charge densities, such as liquids. The moment *l* = 0 corresponds to the net charge in the probe volume and, therefore, vanishes. *l* = 1, 2, 3 correspond to the dipole, quadrupole, and octupole moments, respectively.

The spherical harmonics *Y*_*lm*_(*Θ*,*Φ*) are defined as

17

where  and *P*^*m*^_*l*_ is the associated Legendre polynomial. The spherical harmonics are orthonormal with respect to integration over the unit sphere:

18

We take advantage of this property by expressing the angular dependency of the net charge distribution, Δ*ρ*(*R*_0_,*Θ*,*Φ*)d*R*_0_ on a sphere with radius *R*_0_ = *n*_*R*_*R*_h_ > *R*_d_ and infinitesimal thickness d*n*_*R*_*R*_h_ as

19

The total interaction energy, *U*_de_, describing the interaction of the observation volume with its environment, is given by integrating the product

20 d*U*_de_ = *ϕ*(*R*_0_,*Θ*,*Φ*)Δ*ρ*(*R*_0_,*Θ*,*Φ*)

over the volume outside the probe volume. An integration yields

21

which is an equivalent form of eqn (2).

##### The incomplete Euler Beta function

The incomplete Euler Beta function, *B*_*z*_(*a*,*b*), belongs to the family of generalized hypergeometric functions. The particular case described in this manuscript (*a* = 1 + *λ* > 0) yields the following integral equation:^28^

22

Using the parameters from eqn (10), *a* = 1 + *λ*_*l*_ and *b* = 0, we obtain

23

##### The Lerch transcendent

The Lerch transcendent, *Φ*(*z*,*s*,*a*)^29^ is another function out of the family of generalized hypergeometric functions. For the case described here (*s* = 2, *a* = 1 + *λ*_*l*_), it is characterized by the infinite sum

24

Using the parameters from eqn (10), *s* = 2 and *a* = 1 + *λ*_*l*_, we obtain

25

##### Step by step integration of eqn (8)

To integrate eqn (8) we describe all parameters in the mol fraction frame. Molality is in mol fraction units, described as

26

In mol fraction units, ln *γ*_±_ is given by eqn (4). An evaluation leads to

27

Combining eqn (8), (26) and (27) yields

28

which can be integrated yielding eqn (10)

##### The Debye–Hückel term: dipole–dipole interaction

In the following we investigate in detail the long-range dipole–dipole interaction leading to the equivalent of the Debye–Hückel term in the classical theory of electrolyte solutions. The minimum hydration shell radius *R*_h_ as defined in eqn (12) defines a minimum dipole moment *μ*_0_ = *q*_eff_*R*_h_ inside the observation volume. The composition of the electrolyte determines *q*_eff_ which is expected to be unity for 1 : 1 electrolytes.

In the dilute case, we expect that the average distance between anions and cations will be much larger than *R*_h_. Therefore, we estimate the average distance between anion and cation along the *z*-direction for a given dipole orientation leading to a positive dipole moment along the *z*-direction of *μ*_*z*_ = *q*(*z*_+_ − *z*_−_) = *q*Δ*z* with *z*_+_ − *z*_−_ > 0. To simplify this discussion, we assume a 1 : 1 electrolyte. Anion and cation can take any position within the observation volume so that Δ*z* ranges from 0 to 2*R*_d_. However, the number of ways to realize a dipole with a given strength depends strongly on Δ*z*. For example, there is only a single configuration with Δ*z* = 2*R*_d_ while there are many ways to realize configurations with Δ*z* ≪ *R*_d_. A detailed analysis of the resulting probability distribution shows that the effective distance Δ*z*_eff_ = 0.5*R*_d_ yielding *μ*_eff_ = 0.5*qR*_d_ = 0.5*μ*_0_*R*_d_/*R*_h_. *E.g.* we expect this equation to hold for other electrolytes (*e.g.* 1 : 2, 2 : 1, 2 : 2) when replacing *q* by an effective charge *q*_eff_. For symmetry reasons, this dipole must be located at the center of the observation volume. When an external field *E*_*z*_ along the *z*-axis is applied, the probabilities *P*_p_ and *P*_a_ for an orientation parallel and antiparallel to the electric field, respectively, are different and can be described by Boltzmann's equation. When we assume that  it follows from a linear Taylor expansion that

29

We explore next the dipole induced effect on an ion with charge *q* along the *z*-axis outside the observation volume. The interaction energy *U* of the ion at a position |*z*| > *R*_d_ outside the observation volume with *μ*_eff_ at the center of the observation volume and oriented along the *z*-axis is given by

30

For a positive charge *U*(*z*) is positive above the plane *z* = 0 and negative below. According to Boltzmann statistics, this leads to a depletion of positive charges above the plane and an enrichment below the plane. The expected charge difference between the upper and lower half-plane at the same *z* is given by Δ*q* = *q*(*E*^*U*(*z*)/*kT*^ − *E*^*U*(−*z*)/*kT*^). Integration along the positive *z*-direction assuming |*U*(*z*)| ≪ *kT* yields Δ*q* ∝ *q*^2^*μ*_eff_/*R*_d_. Since *μ*_eff_ ∝ *R*_d_ and the electric field at the center the observation volume *E*_*z*_ ∝ Δ*q*/*R*_d_, it follows that the interaction energy *U*_*μ*,*E*_*z*__ between the dipole inside the observation volume and the electric field generated by the displacement from a single ion in the environment is inversely proportional to the radius of the observation volume. Negative charges will be enriched above the plane and depleted below the plane. The resulting electric fields are additive. The total effect of *ν*_+_ cations and *ν*_−_ anions will therefore be proportional to

31

To relate our description to standard Debye–Hückel theory, we introduce the molecular level ionic strength where  and assume that  and

32

This finding is not restricted to 1 : 1 electrolytes anymore.

Up to now, we have investigated the interaction of our central observation volume with a single dipole along the *z*-axis. To come to a more realistic scenario, we use a second coarse-graining step: we assume that the environment outside the central observation volume consists of dipoles with average dipole moment *μ*_0_ as defined in eqn (32). The central dipole *μ*_c_ interacts with the surrounding dipoles *μ*_s,*i*_*via* dipole–dipole interaction. While perfectly aligned dipoles at a distance *R* close to each other show an interaction energy that scales like

33

the thermally averaged dipole–dipole interaction energy scales like 1/*R*^6^. In general, the number of interaction partners will grow as 4π*R*^2^d*R* with increasing distance *R* from the central dipole.

If we express the distance *R* = *n*_*R*_*R*_h_ between the dipoles in units of *R*_h_ (see eqn (12)) with proportionality constant *n*_*R*_, and normalize the interaction energy by *kT*, we obtain

34

where we have introduced the characteristic length

35

with the reference and where *R*_h_ is defined by eqn (12). The characteristic length and the Debye length *λ*_D_ are related to each other by . *m*^0^ = 1 mol kg^−1^ is introduced formally to yield the proper dimension and corresponds to a hypothetical 1-molal electrolyte solution with no interaction between ions.

##### Concentration dependency of the pre-factor

Both *λ*_c_ and *R*_h_ depend on a parameter combination that is concentration dependent. In the following, we will show that for most typical electrolytes, the concentration dependency is small. We will first focus on the factor . We approximate the dielectric constant as ideal combination of the real parts of the molar susceptibilities, *χ*^∞^_s_ and *χ*_w_ of the solute at infinite dilution and water, respectively:

36

where *c*_s_ and *c*_w_ are the concentrations of solute and water in the solution, respectively, and *V*_0_ = 1 L is the reference volume for concentration measurements. If we further use the average molar volume in the solution

37 *φ̄*_sol_ = *ϕ*_w_ + *x*(*ϕ*_V_ − *ϕ*_w_)

where *ϕ*_w_ and *ϕ*_V_ are the apparent molal volumes of water and electrolyte, respectively, we obtain

38

where Δ*χ* = *χ*^∞^_s_ − *χ*_w_ is the difference in the real part molar susceptibilities of the electrolyte and water, and Δ*ϕ*_V_ = *ϕ*_V_ − *ϕ*_w_ the difference in their apparent molar volumes. At room temperature, the dielectric constant of water *ε*_w_ and its density *ρ* are approximately 80 and 1000 g L^−1^. This yields *χ*_w_ ≈ 1.4 so that . In cases where the term linear in *x* in the denominator becomes noticeable, we can use a Taylor expansion of eqn (37). If the linear term becomes important before the higher order interactions prevail, we would expect an additional contribution in ln *γ*_±_ which scales like *x* × *x*^*δ*^ ln(*x*) = *x*^1+*δ*^ ln(*x*) which is a member of this family of functions with a different exponent. A similar argument holds for the concentration dependency of *R*_h_^2^. In conclusion, in our electrolyte picture, the vivid discussion on how much the change in dielectric constant determines the change in ln *γ*_±_ is meaningless, since a change in dielectric constant and a higher order expansion are equivalent in our description.
